# The Past, Present, and Future of the United States Medical Licensing Examination Step 2 Clinical Skills Examination

**DOI:** 10.7759/cureus.17157

**Published:** 2021-08-13

**Authors:** Jason T Tsichlis, Andrew M Del Re, J. Bryan Carmody

**Affiliations:** 1 Pediatrics, University of Wisconsin School of Medicine and Public Health, Madison, USA; 2 Medical Education, Brown University, Providence, USA; 3 Pediatrics, Eastern Virginia Medical School, Norfolk, USA

**Keywords:** usmle, standardized patient, clinical skills, validity, licensure, medical school

## Abstract

In January 2021, the United States Medical Licensing Examination (USMLE) announced the permanent suspension of their Step 2 Clinical Skills (CS) examination. Launched in 2004, the Step 2 CS examination was intended to ensure that physicians entering graduate medical education possess the necessary information gathering, clinical reasoning, and communication skills necessary to provide patient care. Although the requirement that doctors pass a clinical skills examination as a condition of licensure likely improved some elements of medical education and physician practice, the Step 2 CS examination was deeply unpopular among many medical students since its inception. The demise of USMLE Step 2 CS provides an opportunity to re-examine the test’s value and incorporate improvements in future iterations. However, doing so requires a clear understanding of why the test was so vigorously challenged. Here, we review the history of clinical skills examinations used for medical licensure in the United States and highlight the persistent concerns regarding Step 2 CS’s cost, value, validity, and lack of examinee feedback before proposing future improvements to address each concern.

## Introduction and background

In January 2021, the United States Medical Licensing Examination (USMLE) announced the permanent suspension of their Step 2 Clinical Skills (CS) examination [1]. This decision was necessitated by the novel coronavirus pandemic, which made the administration of a national examination involving standardized patients impractical. Although some medical educators expressed concern about the loss of a standardized measure of bedside skills [2,3], the announcement was widely celebrated by many medical students in the United States, who had long regarded the USMLE Step 2 CS examination as expensive, burdensome, and unnecessary [4-14].

To date, the USMLE has not announced what, if anything, will replace the Step 2 CS examination [15]. For the first time in nearly two decades, doctors can receive a medical license by completing only computer-based examinations of licensure, raising concern about the lack of a national measure of clinical competency [2]. Indeed, careful appraisal shows that the introduction of a nationalized, standardized measure of clinical skills likely improved some elements of medical education and physician practice [16-23]. Yet, since its inception, the value and necessity of the USMLE Step 2 CS examination were vigorously challenged. Here, we review the history of the USMLE Step 2 CS examination; highlight the structural problems with the exam that lead to continued objections from examinees; and suggest solutions to address each of these areas in the future so that any new national clinical skills examination can provide the fairest, least burdensome, and highest value clinical skills examination for medical students.

## Review

How did we get here?

The USMLE Step 2 CS examination was introduced in 2004. However, the requirement that applicants for medical licensure demonstrate practical clinical skills dates back to some of the earliest licensing examinations used by individual states in the late 1800s [24]. Indeed, the first examination created by the National Board of Medical Examiners (NBME) in 1916 included the evaluation of real, not standardized, patients.

To each candidate were assigned three patients, referred to as one long and two short cases. The candidate was given one hour and twenty minutes for the examination of the long case, and required to present a written clinical history, including the physical examination. He was expected further to ask for the laboratory tests required by the nature of the diseased condition, and to stand an oral examination on the clinical history and diagnostic conclusions. The two short cases were accorded twenty minutes each, and the examination was confined to the physical examination of a well-defined condition ... The patients assigned for examination represented the following diseases: typhoid (1); tuberculosis of lung (3); acute endocarditis (1); aortitits with aortic insufficiency (2); aortic insufficiency (1); aortic aneurysm (2); chronic nephritis (1); bronchial asthma (2); chronic myocarditis (1); pernicious anemia (1); diabetes mellitus (2); hypertrophic cirrhosis of liver (1), tabes dorsalis (1); motor hemiplegia (1) [25].

That the NBME chose to include evaluation of practical clinical skills in its original examination is both logical and noteworthy. The NBME was a new organization. To succeed, their examination had to both demonstrate value to test takers and inspire the confidence of state licensing authorities. However, due to its comprehensive nature, including written, laboratory, and oral components, and lasting nearly a week, the NBME’s examination was expensive and burdensome to administer. Registration for the original NBME examination was partially subsidized by nonprofit organizations, but after this funding was exhausted, the price of the examination increased from $5 to $80 (the equivalent of almost $2,000 today) [26,27]. It is perhaps unsurprising that the initial uptake of the NBME examination was modest: in the first decade of the examination’s existence, only 701 examinees received the NBME diploma [25].

Following a commissioned study of British and French medical licensure examinations, the NBME changed to a three-exam format, roughly resembling the content of the three Step examination series in place today [25]. The first two components were written, while the final component aimed to evaluate clinical skills and included a bedside oral examination. By the late 1950s, however, studies of this bedside examination found that performance actually correlated more with the examiner than the examinee [25]. Coupled with rising costs and logistical burdens, the observed clinical examination was discontinued in 1964 [24,25]. For the next 40 years, American medical students were not tested on their clinical skills as part of a licensure examination.

The 1970s saw a dramatic increase in the number of international medical graduates (IMGs) seeking residency training in the United States. By the end of the decade, 21% of all practicing physicians and 20% of resident trainees were graduates of a foreign medical school [28]. In response to residency program directors, who alleged that a significant fraction of IMGs was deficient in clinical skills, the Educational Commission for Foreign Medical Graduates (ECFMG) devised a test, the Clinical Skills Assessment (CSA), intended to determine whether IMGs possessed the minimal skills to begin residency training [29]. In designing the CSA, the ECFMG imposed several boundary conditions, for example, the examination should cost $200 per examinee or less; require an administration time of approximately half a day; and have resources for its administration available at most academic medical centers [29].

Although several other contemporary studies found no differences in performance between United States medical graduates (USMGs) and IMGs [30-33], the ECFMG’s pilot study found that 32% of IMGs scored more than two standard deviations below the mean score for USMGs on the CSA and were considered to have failed the exam [29]. After subsequent studies in 1987 and 1993 found the CSA to be feasible from an administrative standpoint [34,35], the examination became a requirement for IMGs seeking certification for entry to accredited residency training programs in the United States in 1998 [36,37].

The ECFMG’s CSA was generally a success. A survey of program directors found that, following the introduction of the CSA, slightly fewer IMGs were perceived to have started residency with clinical skills deficiencies, with absolute risk reductions of 1-4% across various domains [17]. Over the first several years of its administration, 97% passed the examination; among those who failed, 80% did so due to inadequate spoken English proficiency and/or interpersonal skills [37]. This high pass rate reflected significant self-selection and resulted in a sharp decline in the number of IMGs taking the USMLE and pursuing U.S. residency training. Almost three times as many IMGs took the USMLE Step 1 examination in the two years before the CSA was implemented than in the two years after, and the number of USMLE examinees from non-U.S. and Canadian medical schools has never recovered to pre-CSA levels [38]. According to the ECFMG, the most frequent complaint about the CSA was that it was unfair for USMGs to be exempt from taking such an examination [37].

Following the success of the CSA, the NBME and the Federation of State Medical Boards (FSMB) formally revisited the concept of a clinical skills examination for licensure. The founding documents for the USMLE had noted “the need for and/or desirability of a test of clinical skills” and stated that “when valid and reliable methodologies become available to evaluate such clinical skills, it is anticipated that they will be incorporated into Step 2 and/or Step 3 of the proposed examination” [39].

Initial plans for the clinical skills examination called for it to be administered at 45 testing sites located at medical schools [40]. However, in 2002, the ECFMG and NBME officially agreed to collaborate on the USMLE Step 2 CS, which would be offered at a network of Clinical Skills Evaluation Centers operated as a joint partnership between the FSMB and NBME [41]. The fee for the new examination was set at $975 [42].

The birth of Step 2 Clinical Skills examination and its opposition

The implementation of Step 2 CS was poorly received by medical students and many faculty and administrators. In response to an article introducing the examination [42], a major medical journal published letters to the editor that called the examination “a needless initiative from a self-interested and paternalistic bureaucracy” [5], asserted that the examination’s format “is outrageous, bordering on fraud” [4], argued that “funding [validation studies] with medical students’ money creates a serious conflict of interest regarding the dissemination of any negative findings” [9], and suggested that the test was “so cumbersome, costly, and inconvenient that ... it seems bound to fail” [7]. The Association of American Medical Colleges Council of Deans and the American Medical Association (AMA) also formally expressed concern with the examination [42,43]. Although the NBME and FSMB acknowledged the concerns about the examination, implementing Step 2 CS ultimately required only the assent of the USMLE’s two parent organizations [42]. After the FSMB required passing the clinical skills examination to register for USMLE Step 3, Step 2 CS became a de facto licensure requirement, even without individual state boards explicitly requiring the test [44].

Though licensing authorities and medical educators have generally embraced the examination, many medical students and some faculty continued to express concerns about the value of Step 2 CS [4-14]. Because the percentage of USMGs who pass the Step 2 CS examination on their first attempt is high (and the vast majority of those who do not initially pass do so on their next attempt without any additional preparation), it has been estimated that it costs over $1.1 million to identify a single U.S. student who fails the examination more than once [11]. In 2016, a medical student-led petition to “End Step 2 CS” gathered over 16,000 signatures along with support from the Massachusetts Medical Society and the Michigan State Medical Society [12,45]. The AMA also drafted a policy to work with other stakeholders to transition clinical skills assessment to accredited medical schools [46].

Given the substantial and longstanding opposition to USMLE Step 2 CS, any proposal for a new clinical skills examination seems destined to generate significant resistance from medical students and student organizations. Yet, the reintroduction of a clinical skills examination for licensure might be substantially improved by addressing the specific problems students have cited about the examination since its inception.

Problems and opportunities

Cost

Step 2 CS was expensive. Registration alone cost $1,300 for USMGs and $1,600 for IMGs in 2020 [47,48]. Before the examination’s implementation, the NBME argued that the examination would be overall cost-saving:

The cost of removing physicians from practice is far higher than the cost of preventing them from entering practice. And the cost incurred by the healthcare system and by the patient community is exponentially higher when physicians who have insufficient clinical and communication skills receive licenses to practice medicine [49].

However, if a clinical skills examination saves money for medical boards and the healthcare system as a whole, why should the cost of the examination be borne exclusively by medical students, many of whom are living on high-interest student loans [50]? If the imposition of Step 2 CS provides a financial benefit to other entities, it is both fair and logical that these other stakeholders should share in subsidizing its costs.

The cost of the examination could also be lowered by reconsidering its format. Before announcing its long-term suspension, the USMLE announced plans to administer Step 2 CS using an online telehealth format [15]. The use of such a novel format would require evidence of its reliability and validity but could reduce examination costs for students while allowing the assessment of certain competencies. The USMLE should also revisit the possibility of examinees taking Step 2 CS at their medical school. Although the NBME’s original analysis two decades ago found using regional centers to be the least expensive model [49], most medical schools now manage facilities where standardized patients are employed and well-established objective clinical skills examinations are administered as part of the existing curriculum [16,51].

Even with reduced costs, the NBME could consider offering a no-interest loan or fee waivers and reductions for qualifying examinees. There is a precedent for such a policy. In the 1950s, in response to student concerns about the cost of its examinations (which was then $80 for the entire three-part series), the NBME established a fund that provided interest-free loans to Part III examinees for one year [52].

Lastly, the USMLE should not charge a re-examination fee to examinees who initially fail the examination. By identifying a student whose skills need improvement, a Step 2 CS failure achieves its purpose, and waiving the re-examination fee would help relieve concerns regarding the NBME’s financial conflict of interest in examination policy [53,54].

Value in Residency Selection

It is worth considering why student sentiment toward the USMLE’s Step 1 and Step 2 Clinical Knowledge (CK) examinations differed from the sentiment toward Step 2 CS. Although registration for each of these individual examinations is less costly than Step 2 CS, taking both Step 1 and 2 CK costs essentially the same as Step 2 CS [47,48]. While the consequences of failing any portion of the USMLE can be career-altering, many students perceive that their scores on Step 1 and 2 CK provide the opportunity to improve their standing with program directors in residency selection [55,56]. Yet, because the results of Step 2 CS are reported as pass/fail, the examination can only hurt a student’s residency application [55]. From the student’s perspective as a residency applicant, Step 2 CS provides a stick with no carrot.

To improve Step 2 CS, the USMLE should provide the opportunity for students to distinguish themselves through demonstrating high-quality, real-world clinical skills. This could involve reporting Step 2 CS results as a numeric score, or simply allowing program directors the ability to view an applicant conducting an encounter with a standardized patient [57]. Specialty-specific case vignettes testing skills program directors expect their interns to possess at the start of residency could inspire mastery from students and offer the potential of improving patient care rather than simply screening out perceived incompetence.

The skills tested need not be limited to office-based encounters of common complaints. Although the focus of Step 2 CS has been on evaluating patient communication and information gathering, procedural skills can also be objectively assessed. Indeed, such tests were included in the first NBME examination, where examinees were required to suture a 10 cm segment of the canine small intestine, with the suture lines tested under water pressure [25]. While particular procedural skills may not be relevant to all medical specialties, certain skills, such as those required to respond appropriately to an in-flight medical emergency [58], are common to all physicians and might reasonably be considered a condition of licensure.

Lack of Feedback

Despite the considerable effort undertaken by the NBME to ensure that scores on the clinical skills examination are objective and reproducible, all data used to generate an examinee’s score are kept internally. Examinees receive a pass/fail designation in three broad areas (communication and interpersonal skills, spoken English proficiency, and integrated clinical encounter). This provides examinees with little in the way of actionable feedback to improve performance. Yet, the NBME has historically cited the obligation to inform examinees how their performance compares to the passing standard as a reason to provide scores on their multiple-choice question tests [59].

Providing examinees, even those who pass the examination, with the ability to review specific competencies in which they performed well and where they should devote time to improving has a well-established rooting in learning theory and has long been considered characteristic of a sound assessment by education theorists [60]. To maximize the value of this feedback (and minimize the potential for secondary use), more detailed commentary could be provided to examinees through their personal USMLE portal but not included on the USMLE transcript.

Validity

Though there is likely widespread agreement among students, testing organizations, licensing authorities, and the general public that the clinical skills examination should provide a valid assessment of a physicians’ capabilities, there has been less agreement on what type of validity evidence should be required. In 2004, concerns that Step 2 CS was being implemented without having demonstrated predictive validity were brushed aside:

There is, however, limited evidence that any examination, including the existing components of the USMLE, predicts long-term outcomes ... [T]here is no precedent for requiring proof that a test predicts the future performance of a student, and the public has little patience for such an intellectual debate [42].

The NBME has since published two studies that demonstrate a modest correlation between Step 2 CS data interpretation scores and communication/interpersonal skills scores and similar assessments of internal medicine residents [18,23]. However, the studies found no correlation with measured data-gathering ability, and concerning communication scores, the association was admittedly “relatively weak” [23].

Similarly, even after over 15 years of testing, little evidence for construct validity has been established. Interestingly, following the suspension of USMLE Step 2 CS in spring 2020, the ECFMG began to require the Occupational English Test (OET) as a condition of certification [61]. The ECFMG’s president subsequently noted that pass/fail rates for the OET “fall in the same range as Step 2 CS” [62], suggesting that the examination functioned largely as a test of spoken English proficiency rather than a robust measure of clinical skills.

Instead, proponents of Step 2 CS have argued that the examination provides face validity [42,63]. It is logical for the public to expect licensing authorities to require a measure of clinical competence, and this requirement may inspire confidence in patients and physician self-regulation. Other supporters of Step 2 CS have argued that the examination has consequential validity based on improved curricular time and attention devoted to clinical skills education [19,64].

Before any new clinical skills examination is implemented, the test maker should seize the opportunity to engage stakeholders in a discussion about how best to establish that a clinical skills examination measures what it should. What evidence should we require, and how should that evidence be sought? Careful studies with endpoints defined by consensus should be initiated when the test returns. If those endpoints are not achieved, the test should be revised to meet them.

## Conclusions

It could be argued that Step 2 CS was a successful examination. Evaluating physicians based upon their demonstration of clinical skills, not just their performance on written tests, was logical and provided reassurance to the public. Furthermore, the existence of a clinical skills examination improved clinical skills education in medical school, and the examination itself may have prevented a small number of incompetent practitioners from entering practice. Yet, even Step 2 CS’s staunchest defenders must concede that the examination was never able to overcome long-simmering concerns about its cost and validity. We believe the reasons for student and administration resistance to Step 2 CS were legitimate, expressed in good faith, and must be addressed in any future clinical skills examination. The termination of USMLE Step 2 CS provides an incredible opportunity to revisit the structure, function, and necessity of a national clinical skills examination, but any improvement requires that we learn from the experiences of the past.
